# The reciprocal jaw-muscle reflexes elicited by anterior- and back-tooth-contacts—a perspective to explain the control of the masticatory muscles

**DOI:** 10.1038/s41405-020-00056-z

**Published:** 2020-12-17

**Authors:** Lauri Vaahtoniemi

**Affiliations:** Plusterveys Hammasateljee, Kokkola, Finland

**Keywords:** Mandibular muscles, Dental anthropology, Occlusion

## Abstract

**Aims:**

Tooth-contact sensations are considered essential to boost jaw adductor muscles during mastication. However, no previous studies have explained the importance of the inhibitory reflex of human anterior-tooth (ANT)-contacts in mastication. Here I present the “reciprocal reflex-control-hypothesis” of mammalian mastication.

**Subjects and setting of the study:**

I demonstrate the hypothesis with the live kinematics of free jaw-closures as inferred from T-Scan recordings of dental patients.

**Results:**

The jaw-closures started with negligible force, predominantly with ANT-contacts (the AF-bites). The first ANT-contact inhibited the first kinematic tilt of the mandible, whereas the bites starting from a back-tooth (BAT)-contact (the BF-bites) accelerated the first tilt. The second tilt established a low-force static tripod of the ANT- and bilateral BAT-contacts for a fixed mandible-maxilla relation. Thereafter, semi-static bite force increased rapidly, relatively more in the BAT-area.

**Discussion and Conclusions:**

In the vertical-closure phase of chewing, the primate joint-fulcrum (class 3 lever) conflicts with the food-bolus-fulcrum in the BAT-area (class 1 lever). The resilient class 3 and 1 lever systems are superseded by an almost static mechanically more advantageous class 2 lever with a more rigid fulcrum at the most anterior ANT-contact. For humans, the class 2 levered delivery of force also enables forceful horizontal food grinding to be extended widely to the BAT-area.

## Introduction

From studies comparing the chewing capacity of natural, and implant-supported dentitions, it has been concluded that the periodontal mechanoreceptor (PMR) -elicited added muscle activity is essential to enhance human masticatory performance.^1^ Mammalian PMRs share a common basic element: they respond to stretching.

The microanatomic ultrastructure, neural connections and functions are different for different types of teeth and between animal species with different types of mastication.^2,3^ Rabbits’ molar-tooth-contacts elicit rapid excitation of the masseter muscle^4^ and the PMRs of the cat canine-tooth elicit equally rapid masseteric activity, within a few milliseconds (ms) of a tap-contact.^5^ But in contrast in humans a rapid inhibition of temporal and masseter muscles is evoked by a tap-, or a push-contact of the central incisor. The latency of this inhibitory reflex is about 10 ms and it lasts some dozens of ms, after which the temporal and masseter muscles resume their activity.^6,7^ Evidence from dental patients suggest that during lateral, gliding excursions of the mandible, the canine tooth contacts, akin to the incisor contacts, inhibit and switch off the activity of the temporal and masseter muscles.^8^ In contrast, mechanical stimulation of the human upper first molar rather causes immediate excitation, not inhibition, of the masseter and temporalis muscles.^9^ Williamson and Lundqvist (1983) concluded, that the BAT-contacts of mastication rapidly boost the masseter and temporalis muscle activity.^10^

During the closure phase of the cercopithecoid chewing cycle, the inferior belly of the lateral pterygoid muscle remains silent while the temporal and masseter muscles are active.^11^ The human medial pterygoid muscle has also been shown to be reciprocally silent during the activity of the temporal-masseter complex.^12^ By the end of the vertical phase of the unilateral closure movement, the path of the human mandible turns into a horizontal movement towards the midline.^13–15^ The horizontal phase of closure is possibly driven by the working side medial pterygoid muscle,^16^ and the inferior belly of the lateral pterygoid muscle.^17,18^ A general agonist-antagonist relation between the vertically (the temporal-masseter-complex), and the horizontally oriented jaw-adductor muscles (the inferior belly of the lateral, and the medial pterygoid muscles) may be assumed.^12^

A tap or a push-contact of an incisor tooth generally induces the inhibition of the masseter muscle at first, followed by later excitation of the masseter muscle.^6,7^ Variable inhibition-excitation reflex response patterns of masseteric activity have been explained to depend on the force and the duration, and the rate of rise of stimulation. The dualistic outcome of the mechanical tooth-contact stimuli has been explained by the different neural conduction velocities between anatomically different PMR-receptor populations.^19^ The PMR-afferent nerves are unique in that they present two alternative pathways. The cell bodies of the PMR-afferent nerves originate either from the trigeminal ganglion (TG) or, different from all other peripheral sensory nerves, they have an alternative route from the trigeminal mesencephalic nucleus (MN) in the central nervous system (CNS). Both types of PMRs, with TG- and MN-connections are encountered in the same tooth, but the proportions of these distinct PMR-populations differ between the apical and coronal part of the tooth root.^3^ An axial tap-stimulus is likely to stimulate most the apical area PMR-populations. An orthogonal tap-stimulus is likely to cause opposite stimuli for the PMRs located on different sides of the mechanical fulcrum around which the tooth root tilts in its socket. Accordingly, the inhibitory synaptic potentials have been suggested to be induced by the fast-connection PMRs while the slow-connection PMRs should be responsible for the later, excitatory activity^19^ However, there are no previous studies in which the reciprocity of the ANT- vs. BAT-contact elicited reflexes would have been considered.

**Fig. 1:**
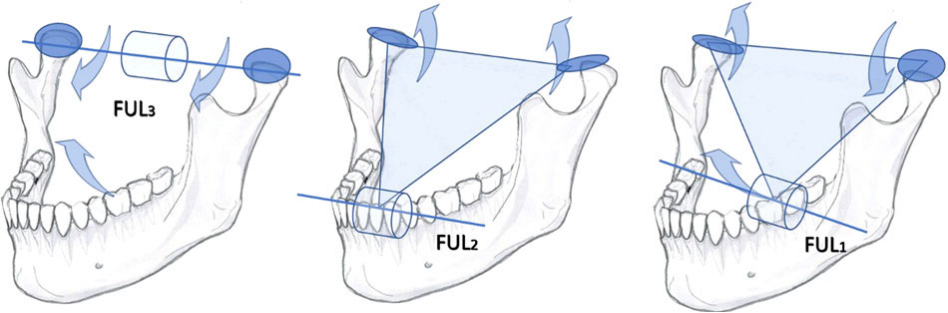
The possible kinematics of mandible in a free, vertical jaw-closure. In the left panel, the condylar axis serves as a fulcrum (FUL_3_) for a class 3 lever for the clockwise rotation of mandible (arrows). The anterior part of mandible moves upward. Minimal amount of reaction force is subjected to the temporomandibular joints, and the synovial spaces of the joints are presented as inflated circles. The middle panel depicts the instant of the first tooth contact of an AF-bite. The unyielding ANT-contact stops the original movement. The point of contact instantaneously creates a novel fulcrum (FUL_2_) for a class 2 lever, to causes an acute counterclockwise tilt of the body of mandible. The synovial fluid spaces of both jaw joints are presented to be deflated and flattened by the reaction force. The right panel illustrates an alternative course of the kinematics of mandible, where a BAT-contact occurs first (the BF-bite). The first contact in the left BAT-area instantaneously transforms into a fulcrum (FUL_1_) for a class 1 lever. The reaction force tilts the tip of mandible upward, whereas distraction of the ipsilateral joint (inflated circle), concomitant with compression of the contralateral joint (deflated circle), may be expected.

In this study the teeth of the human dental arches were dichotomised to belong either to the “immediately inhibitory ANT” (the incisors and canines), or the “immediately excitatory BAT” (the premolars and molars). My study hypothesis is that the PMR-mediated sensory inputs begin from the instant when the mandible-maxilla collision forces are first subjected into a single bite point, the first tooth-contact of dental occlusion. The kinematic consequences for mandibular movements following the first tooth contact were first presented by Greaves.^20^ I suggest three alternative mandibular kinematic patterns each with a distinct force leverage system for the free vertical jaw-closures (Fig. 1).

While chewing the lever system to be deployed depends on the instantaneous resiliency of the food bolus. A soft piece of food may be crushed almost entirely by the original class 3 lever with the temporomandibular joint (TMJ)-area as its fulcrum (Fig. 1, left panel). However, a tough piece of food in the BAT-area would transform the mandible into a class 1 lever (similarly as in Fig. 1, right panel) with its fulcrum at the more or less resilient bolus. The ambiguous conflict between the shifting fulcrums of the classes 3 and 1 lever systems resolves as soon as the class 1-levered anterior part of the mandible tilts upwards to enable a more rigid fulcrum at ANT-contacts (Fig. 1, middle panel) for a class 2 lever to supersede the previous class 3 and 1 lever systems. The jaw-adductor muscles should respond, within milliseconds, to any of these alternative instantaneously changing lever systems of mastication.

The T-Scan is a clinical diagnostic tool for measuring the force and timing of tooth-contacts developing in the different parts of the dental arch,^21^ offering three-dimensional, in vivo -recorded data of the kinematics of mandible during the tooth-guided phase of dental occlusion. Koos et al.^22^ used the T-Scan to follow the incremental build-up of tooth-contacts of “normal” subjects, in consecutive 10 ms time intervals, starting from the first contact of occlusion. For 40% of the bites of the study the first contact of occlusion happened in the ANT-area (the AF-bites), whereas the bites with the first tooth-contact in the BAT-area (the BF-bites) were most prevalent (44% of the recorded bites). A slightly high dental restoration, or misaligned teeth unilaterally in the BAT -area are not uncommon findings for an average dental patient, that may have predisposed for the likelihood for the BF-bites to occur. The rest, 16% of the recorded bites of that study had simultaneous first contacts in both ANT-, and BAT -areas (the SIM-bites) during the first 10 ms of the recorded bite.^22^

Comparing the T-Scan-recorded kinematics of the AF-bites (initiated by the plain ANT-contact stimuli) to the kinematics of the BF-bites (initiated by the plain BAT-contact stimuli) should enable a natural experiment-setting to evaluate the assumed reciprocity between the ANT- and BAT-evoked reflexes. The reflexes elicited by the AF-bites (Fig. 1, middle panel) should, at first, cause inhibition of the masseter and the temporal muscles, i.e. a subdued kinematic response of the mandible. In contrast, for the BF-bites (Fig. 1, right panel) a rapid, reflex-activation of the masseter and temporal muscles, and thereby a rapid kinematic movement of the mandible should be expected.

In the present study I make the assumption of reciprocity of the immediate reflex actions between the ANT- or BAT-contacts of mastication to explain the selective activation of the antagonistic vertically oriented temporal-masseter- muscle-complex, and the horizontally oriented complex of the inferior belly of the lateral pterygoid and of the medial pterygoid muscles.

## Subjects and methods

Patient data were obtained from 48 subjects, 30 women and 18 men, to whom I had performed a T-Scan recording as a part of dental diagnosis and treatment in my practice. The age of participants varied from 22 to 72 years. The patients were individuals without crossbites or any known medical disturbances that could be assumed to limit the coherence of their normal jaw-muscle function. Most patients had variable signs and symptoms of possible TMJ-related problems. When examined in the maximum intercuspal position, all subjects had at least one natural occluding tooth and no more than one dental implant per each upper and lower, left and right set of molars, premolars and incisors. None of the implants were canines, or without an adjacent natural tooth of the same set. I made no attempts to categorise the study subjects according to their dental, or TMJ health status.

### Technique

The T-Scan system comprises of a mylar foil sensor embedded with a matrix-grid of columns and rows of pressure-sensitive electronic ink. The horseshoe-shaped area of pressure-sensels covers the occlusal-contacting areas of dental arches. The location, force and timing of a contacting tooth is recorded to be presented graphically on the computer display. A T-Scan Novus, version 9 sensor foil (Tekscan Inc., South Boston, MA 02127, United States), large or small, was selected to match each patient’s dental arches. For each recording session, the software-wizard-guide was used to adjust the fitting of the sensor and voltage for individual occlusal anatomy and bite force, according to manufacturer’s instructions. The recordings were taken from subjects sitting upright in a simple table chair, without a headrest. They were instructed to imitate a slightly forward inclined upright head position, that they would feel comfortable for chewing and swallowing normally, as at a dinner table. I placed the sensor against the maxillary teeth with the pointed tip of the sensor-holder between the upper incisors, according to the manufacturers’ instructions. With the sensor sheet between their teeth, the subjects were instructed to close their mouth normally, until they felt their teeth comfortably seated in a stable closed position.

### Retrieval of data from T-Scan display

The T-Scan software records the build-up of tooth contacts of jaw-closure in “T-Scan movie”-frames propagating in three ms intervals. The T-Scan displays the time elapsed and the bite force at the given timepoint, expressed as the percentage of the maximum force of each particular recording (the mFpr). In addition, a schematic upper dental arch is displayed on the computer screen to indicate to which teeth the contacts are building up, and by which percentage of the instantaneous mFpr each tooth is loaded. I trichotomized the upper dental arch into the ANT-area comprising the central and lateral incisors and the canines, and the rest of the dental arch as the left and right BAT. The widths of the central, and lateral incisors and the canines were assumed similar for all subjects: 8.5, 6.5 and 7 mm, respectively, according to the T-Scan software default, and the rest of the arch as left, or right BAT.

The “close-hold” -part of the data from the clinical T-Scan examination protocols (the right and left lateral excursions, and the Multi-Bite-procedure^21^) was manually extracted, frame-by-frame, from the recorded T-Scan movie into a spreadsheet. The three repeated recordings/subject, included in the clinical examination protocol, were performed in a single visit, within minutes of each other. Between the recordings, no interventions were made to the dentition.

### Data-analysis

The bites with a first ANT-contact were named AF-bites, whereas bites starting from a BAT-contact were named BF-bites. The bites with simultaneous first contacts in the ANT- and BAT- areas (within the three ms time-frame of the T-Scam -movie) were named SIM-bites category.

The timepoints and the study parameters are presented in Table 1. Differences of the timing- and force- parameters were compared between the AF- and BF-bite-categories. The proportion of force subjected to the ANT in relation to the BAT (the ANT/BAT-ratio) was calculated for each timepoint. The SIM-bites in this study were excluded from the statistical ANT/BAT comparisons. The statistical analysis was retrieved 2020, April, from https://www.socscistatistics.com/tests/.

**Table 1:** The study parameters.

1. *The timepoints and the force parameters of this study*
tZ – the time-point of the first, uninterrupted, anterior area tooth contact of each bite, leading to a complete occlusion in all parts of dentition.
tW – the time-point of the first, uninterrupted, left or right back-tooth contact of each bite, leading to a complete occlusion in all parts of dentition.
The FCf -force parameter indicates the percentage of the bite force at the first contact of occlusion (FC), in relation to the maximum bite force of each particular recording (mFpr).
The FTf -parameter indicates the mFpr at the timepoint of the accomplishment of the first tilt of occlusion (FT).
The tZ was the FC of the FC of AF- and SIM-bites, and the FT of the BF-bites.
The tW was the FC of BF-bites, and the FT of the AF- bites.
tS – the timepoint when, for the first time, simultaneous contacts were established in the ANT- and bilateral BAT -compartments of dentition.
The STf -parameter indicates the mFpr at the accomplishment of the secondary tilt (ST) for the stabilising triangle of support for both AF- and BF-bites.
t1 – the time-point closest to when 1% of the mFpr had been established.
The t1f -parameter indicates the mFpr at t1.
t10 – the timepoint closest to when 10% of the mFpr had been established.
The t10f -parameter indicates the mFpr at t10.
tB – the time-point generated by the T-Scan computer program algorithm, at which the “maximum amount of contact points” had been attained.
The tBf -parameter indicates the mFpr of the tB.
t95 – the time-point closest to when 95% of the mFpr had been established.
The t95f -parameter indicates the mFpr at t95.
2. *The ANT/BAT ratio of force*
The combined percentages of mFpr forces subjected to each individual tooth of the ANT-area in relation to the combined percentages of mFpr subjected to each individual tooth of the left and right BAT-compartments of the upper dental arch.
3. *The timing-parameters of this study*
The FTt -timing parameter indicated the duration of time elapsed (ms) for the FT.
For the AF-bites The FTt indicated the time elapsed from tZ to tW.
For the BF-bites, the FTt indicated the time elapsed from from tW to tZ.
The t1t -timing parameter indicated the duration of the initial, very low bite force (<1% of the mFpr), starting from the FC until t1.
The STt -timing parameter indicated the time required for the establishment of a triangle of tooth contacts between ANT and bilateral BAT-compartments.
For both AF- and BF-bites the STt indicated the time elapsed from FC until tS.
The t10t -timing parameter indicated the time elapsed from FC until t10.
The tBt -timing parameter indicated the time elapsed from FC until tB.
The t95t -timing parameter indicated the time elapsed from FC until t95.

### Limitations of the technique

The T-Scan is a hand-held measuring tool. Clinical skills and routine are essential to minimise operator bias. Occasionally, prior to the first continuous tooth contact, repeated tap-like contacts, with a few ms of an interval between the taps, were observed. These contacts were considered artefactual and ignored in the assessment of the time-point of the first tooth contact (tZ or tW). Regular bite foil (Trollfoil, Pre-mounted Articulating Foil 8 Micron, TrollDental Sweden AB, 46153 Trollhättan, Sweden) was used to visualise and compare the localisation of occlusal contacts with T-Scan data. However, it was not always possible to definitively ascertain, whether the T-Scan data of contacts was coming from the distal aspects of canines or from the mesial aspects of the first premolar teeth.

### Ethical considerations

The T-Scan is a non-invasive diagnostic tool to be implemented in the clinical examination of dental patients. The study protocol per se did not intervene or alter the health status of the patients. I informed and obtained the consent and permission from my patients, that their T-Scan data could eventually be used for publication purposes. The study protocol was presented to the Regional Ethics Committee of the Northern Ostrobothnia Hospital District, Oulu, Finland, and it was granted and approved to conform to the regulatory norms.

## Results

Data from the three successive bite-recordings from 48 patients, produced 144 recorded bites. Out of the three repeated bites, 26 subjects had AF-bites only, whereas three individuals consistently had BF-bites only. Altogether, 112 AF-bites (77.7%), and 26 BF-bites (18.1%) were selected for analysis. Six SIM-bites (4.2%) were found in five subjects. As examined by Kolmogorov–Smirnov tests, none of the force parameters (the FCf, FTf, tSf and at tBf) and none of the timing parameters (the FTt, STt, t1t, t10t, tBt and t95t) were normally distributed. However, the general patterns of timing and force development of tooth-contact build-up were individually characteristic. The Spearman’s Rho calculator showed a statistically significant within-individual correlation between the three repeated recordings for all of the parameters of this study. Similar within-individual scores were obtained for the three, repeated bite-recordings, as indicated by the non-significant results of the Friedman Test for Repeated Measures.

The mean percentages of the mFpr and the s.d., and the min and max of the force parameters (FCf, FTf, t1f, STf, t10f, tBf and t95f) of the AF- and BF-bites are shown in Table 2. The SIM-bites were excluded from the statistical comparisons of the study parameters. The force parameter scores did not differ significantly between the AF- and BF-bites as tested with one-way ANOVA. The decline of the ANT/BAT -ratio of force indicated the bite force to increase relatively more in the BAT area for both AF- and BF-bites throughout the course of the jaw-closure process.

**Table 2:** The force parameters.

The values the force parameters and the ANT/BAT ratio are given in % of the mFpr						
	AF-bites			BF-bites		
Parameter	Mean (s.d.)	Min–max,	Ratio	Mean(s.d.)	Min–max,	Ratio
FCf	0.3 (0.2)	0.1–0.9	100	0.2 (0.2)	0.1–0.7	0
FTf	3.0 (3.3)	0.2–20.7	4.3	2.3 (4.7)	0.2–15.9	0.5
t1f	1	1	6.9	1	1	0.6
tSf	7.6 (8.8)	0.4–43.6	2.9	4.0 (4.6)	0.5–15.9	0.8
t10f	10	10	1.8	10	10	0.8
tBf	77.9 (7.3)	57.3–92.0	0.5	77.2 (9.2)	43.4–93.7	0.4
t95f	95	95	0.4	95	95	0.3

The axis between the ANT- and the unilateral BAT-contacts created by the first tilt then tilted over to the contralateral BAT-direction (the second tilt). At the timepoint tS, a tripod of bilateral BAT- and ANT-contacts was created. For five BF-bites from three subjects, a tilt to the contralateral BAT-contact occurred before there were any ANT-contacts. However, these five, exceptional BF-bites presenting simultaneous tS and tZ, were included in the calculations as “normal” BF-bites.

The mean, s.d., and the min and max of the timing parameters are given in the Table 3. The FTt-parameter of the BF-bites proceeded significantly faster (mean, 24 ms, ±s.d. 17) than that of the AF-bites (mean, 66 ms, ±s.d. 61), as compared by one-way ANOVA [*F*(1, 272) = 6.91445, *p* = 0.009037]. For the rest of the timing parameters, no significant differences between the AF- and BF-bites were found.

**Table 3:** The timing parameters.

The durations of the timing parameters are given in ms, and compared with ANOVA					
	AF-bites		BF-bites		
Parameter	Mean(s.d.)	Min–max	Mean (±s.d)	Min–max	
FTt	67 (61)	2–332	24 (17)	2–71	^a^
t1t	52 (53)	0–231	43 (39)	0–154	N.S.
STt	113 (102)	6–605	74 (76)	2–357	N.S.
t10t	151 (124)	14–738	138 (116)	16–411	N.S.
tBt	385 (284)	56–1817	334 (238)	115–928	N.S.
t95t	640 (416)	118–2529	584 (462)	198–2497	N.S.

^a^Significantly different as compared by one-way ANOVA [*F*(1, 272) = 6.91445, *p* = 0.009037].

## Discussion

### The “ANT is not BAT” -dichotomy of PMR sensory inputs

According to the main hypothesis of this study, the “reciprocal reflex-control-hypothesis”, a sensory signal is required from BAT-contacts for the temporal and masseter muscles to activate, whereas ANT-contacts cause an immediate inhibition of the same muscles. It is intriguing to speculate, which muscles lifted the mandible before any tooth contacts. Tooth contact-elicited PMR-signals seem not to be required for the medial pterygoid muscle to activate.^23^ It may be that weak activity of the bilateral medial pterygoid muscles exclusively started to lift the mandible from its resting position. The first tooth-contacts were always negligibly weak for both AF- and BF-bites, well below 1% of the mFpr (Table 2). The force to initiate the free jaw-closure process was just enough to overcome the gravity and the viscoelastic resistance of the masticatory apparatus. Perhaps, the bilateral low-force activity of the medial pterygoid muscles are the principal agonists to gently start the free jaw-closures, whereas the temporal-masseter-complex is the source of power reserved for the later, almost static, but much more forceful, vertically oriented jaw-closure. In conclusion, the temporal-masseter-complex was deployed by two alternative ways. Either immediately after the short latency period of the excitatory reflexes elicited by the BAT-contacts of the BF-bites (the mean duration of the FTt being 24 ms, ±s.d. 17), or in a delayed manner, after the latency and inhibitory sensory inputs from the ANT-contacts of the AF-bites had waned (with the FTt mean 66 ms,±s.d. 61). The between-individual durations of the timing of the kinematic events (Table 3), and the force parameter scores of the three, repeated bite-recordings varied widely (Table 2), and they were not normally distributed. The dental patients of this study each presented an individual variation of dental arch anatomy and degree of laxity of their TMJs. Furthermore, it was not always possible to definitively ascertain the ANT-BAT -dichotomy. Perhaps, a tooth-contact, as displayed by the T-Scan to originate from the mesial aspect of a first premolar, was really coming from the distal aspect of the adjacent canine tooth. The results for the FTt-parameter can be interpreted that depending on which one of the two reciprocal reflex mechanisms occurs first determines the kinematics of the free jaw-closures. The sensory outputs from the plain BAT-contacts initiating the BF-bites elicited a rapid first tilt of mandible, whereas the plain ANT-contact inputs initiating the AF-bites caused a temporary stall of the first kinematic tilt of mandible.

### The instantaneous locations of mandibular condyles in a free jaw-closure

The resultant force vector of bilaterally active medial pterygoid muscles is slightly protrusive, but possibly more importantly, the preponderance of the AF-bites of this study (77.7%) reflects the forward translating abilities and the resiliency of the human TMJ as a rotational fulcrum. Opening gape was required for the manoeuvring of the T-Scan sensor into the mouths of the subjects. Mouth-opening translates the condyles forward, inflating those synovial compartments of the human TMJ that are posterior and superior to the condyle head,^24^ and distract the mandibular condyle from the confines of the slopes of the articular eminence, downwards in the glenoid space. Perhaps, in analogy to the conclusions drawn by Huddleston Slater et al.,^25^ the centre of rotation of mandible was probably hanging low and protruded in the glenoid space at the start of the T-Scan recording of this study. During the initial, low-force closure movement of the mandible, still without any tooth contacts yet (Fig. 1, left panel) the reaction forces were probably not enough to deflate the synovial aqua bed of the glenoid fossa, adding the likelihood for the AF-bites to occur.

The slightly forward inclined head-posture, as instructed to the subjects of the present study sitting on a stool and unsupported by a headrest may also have added to the percentage of AF-bites in comparison to the otherwise analogous T-Scan recording technique employed by Koos et al.^22^ For ventrally flexed head positions, the axis of rotation of mandible moves forward, according to the “sliding cranium” -phenomenon^26^ possibly increasing the likelihood of AF-bites, as has been demonstrated in previous T-Scan studies.^27,28^ Furthermore, tiny, jaw-closure associated ventral head movements of the unsupported head are coined with protrusion-oriented movements of mandible.^29^ During the natural, cyclic chewing movements, the repeated open-close movements would probably not allow sufficient time for the synovial spaces to recuperate and re-inflate between cycles. In my not yet published study the second, immediately repeated open-close cycle of the “Multi-Bite” recording protocol, showed the cyclically repeated “second bites” to proceed with significantly shorter FTt, and being more predominantly of the BF-bite category. Thus, the cyclically repeated chewing movements appear to flatten the synovial spaces of the TMJ and alter the kinematics of jaw-closures by shifting the location of the instantaneous axis of rotation of the mandibular condyles to the posterior and superior direction.

### The “triangle of support” for the semi-static phase of free jaw-closures

**Fig. 2:**
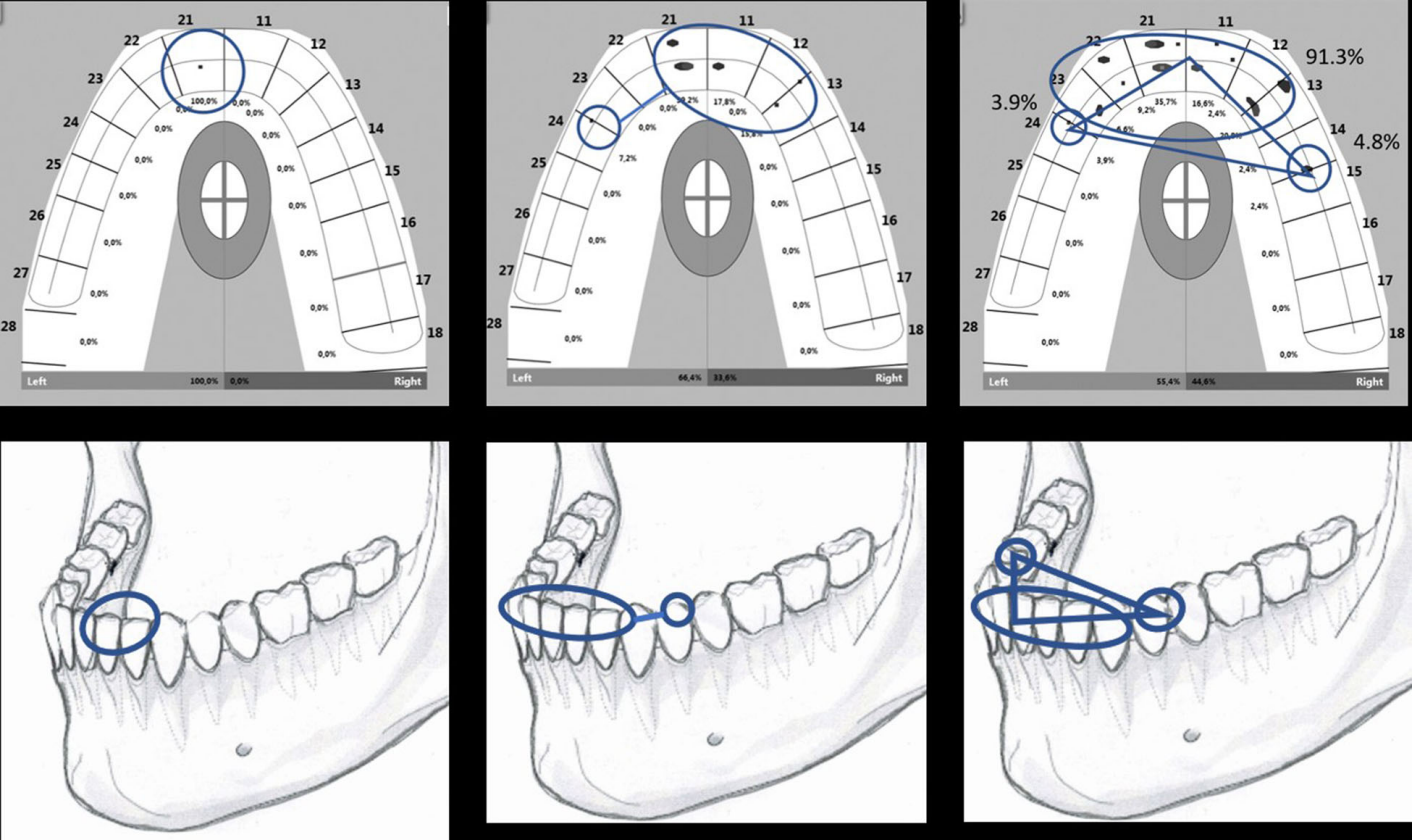
The kinematics of a typical AF-bite. Succession of screenshots of a T-Scan movie demonstrate the development of occlusal contacts of the upper dental arch from timepoint tZ until tS. For each tooth the percentage of the bite force of that timepoint is indicated. The left panel depicts the timepoint tZ, the starting point (0 ms) of accumulation of occlusion contacts. The total bite force is 0.2% mFpr, of which 100% is subjected to the left central incisor. The ANT/BAT ratio of force distribution is 100/0. The middle panel is a snapshot taken 53 ms later, at the timepoint tW. The total bite force is 2.0 % of the mFpr. All the ANT-contacts together bear 92.8% of the mFpr, whereas the rest, 7.2% of the bite force is subjected to the BAT, the left first premolar. The left BAT and the multiple contacts of the ANT area (encircled) form an ANT-BAT-axis allowing for a contralateral tilt of mandible only. The right panel, at timepoint 102 ms after the first contact of the occlusion process, depicts the “triangle of support” -concept. The occlusal contact forces are distributed to the left BAT, ANT and the right BAT compartments of the dental arch. The bite force is 6.1 % mFpr. The ANT/BAT ratio is 91.3/8.7.

**Fig. 3:**
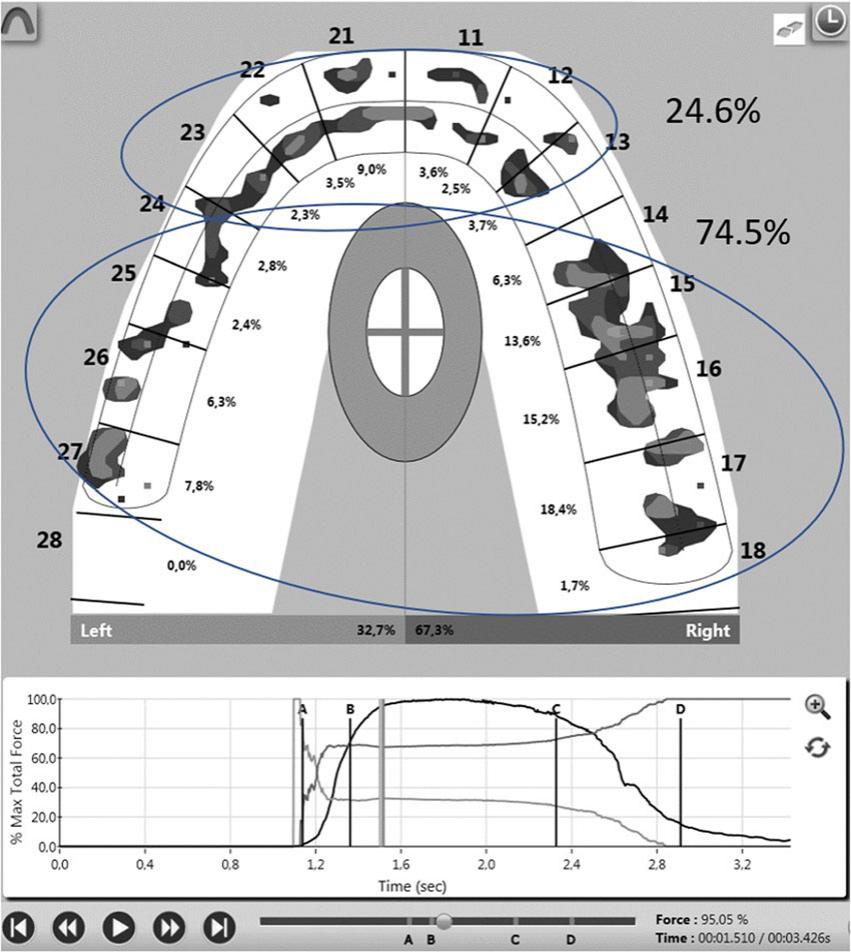
The same T-Scan recording as in the Fig. 2. at t95. The force/time graph, the mFpr and the time elapsed are also displayed. The bite force is 95.05% mFpr. The ANT/BAT ratio is 24.6/74.5.

The first tooth contacts remained almost stationary. The reaction forces of the mandible-maxilla collision were eliminated by the resiliency of the periodontal tissues, bones and joints. The t1t-parameter of this study (during which the almost static bite force remained at <1% of the mFpr) was approximately equally long for both the AF- and BF-bites (Table 3). The first ANT contact of an AF-bite (Fig. 2, left) stopped the dynamic upward movement of the mandible, and the original class 3 lever ceased to exist. A class 2 lever now superseded, with the novel ANT-contact as its fulcrum, to deliver the muscle force for an oppositely directed tilt-like movement of the body of the mandible (Fig. 1, middle panel). Gradually increasing numbers of low-force tooth contacts started to develop in other parts of the dental arch to witness the first tilt of mandible, the duration of which was the FTt-parameter of this study. For the BF-bites, the first tilt was of different nature, opposite in its direction compared to the AF-bites. The first BAT-contact of the BF-bites served as a fulcrum for class 1 levered upward movement of the mandible (Fig. 1, right panel), causing an accelerated first tilt of the tip of mandible upward, for ANT-contacts. As a result of the first tilt, either of the AF-, or of the BF-bites, an axis between the ANT- and the unilateral BAT- compartment of the dental arch (Fig. 2, middle) limited the degree of freedom for the forthcoming tilt-like movements of mandible and only one direction for the subsequent tilt-like movement remained possible. The second tilt of the mandible to the contralateral BAT-contacts finalised the triangle of support between the ANT- and the bilateral BAT-contacts. Thereafter, identically for the AF- and BF-bites, the tripod of contacts, analogous to a three-legged stool, prevented the future tilts of the mandible-maxilla-relations (Fig. 2,c). The number of tooth-contacts started to increase suggesting semi-static yielding of the teeth and supporting tissues in response to the rapidly increasing force. The ANT-area was bearing relatively more bite force at first, whereas later, approaching the complete intercuspal position, most of the static bite force was accumulated in the BAT-area (Fig. 3). Table 2 shows the ANT/BAT ratio diminishing towards the endpoint of the recordings, in accordance with the findings of Koos et al.^22^ It may be concluded that for the human masticatory pattern most of the increase of the masticatory force is delivered by an almost static class 2 lever after the establishment of the triangle of support with its most anterior ANT-contact as its fulcrum.

### The neuroanatomy of chewing, fast and slow

The T-Scan-method presented here enabled the comparisons of kinematic reaction-effects between the stimuli from two anatomically distinct sources, the ANT or BAT. The study hypothesis assumed the ANT-PMR-populations and neural connections are different from the respective pathways and inputs of the BAT-area to explain the qualitatively opposite muscle reactions, fast and slow. When whole, uncooked carrots are on the *menu* we have two modes of chewing activity. We break the carrot between our ANT with a cautious and slow chewing stroke and continue chewing the morsels in the BAT-area with fast chewing strokes. Or, we can put the tip of carrot directly between the BAT and start the fast chewing right away.

The PMR-afferent nerves are unique in that their sensory information is delivered by two alternatives, the TG- and MN- pathways. Those cell bodies of the PMR-afferent nerves that originate from the MN differ from all the other peripheral sensory nerves by having a direct route to the CNS. The rationale for these two distinct routes for the afferent pathways of the different PMR-populations is not perfectly understood, but it is possible that the PMR-afferents with cell bodies in the MN have a different functional role than those of the TG.^30^ Perhaps, the dual pathway system exists to separately deliver the fast and the slow excitatory reflexes. The cat canine tooth is abundant with PMR-connections to the MN.^30^ These connections have been shown to be excitatory,^5^ which has been speculated to serve the predatory functions of the canine tooth for the *Felidae* family.^31^ The monosynaptic connections via MN do not involve inhibitory interneurons and might be considered fast activators, whereas the polysynaptic sensory afferents taking their route via TG would possibly be modulated by inhibitory interneurons to slow down muscle activation. Whether the human BAT-MN or ANT-MN or the respective -TG connections are inhibitory or excitatory can only be speculated. The neural connections of cercopithecoid incisors and canines have more connections with MN and TG than molars and the relative abundance of TG-connections for ANT as compared to BAT-connections “may impart greater sensory perception” for the ANT, whereas the BAT connections might be responsible of the “initiation of complex jaw reflexes where large occlusal forces are generated” as speculated by Hassanali.^2^

The spindle-elicited activity of temporalis and masseter muscles sets on within milliseconds of a stretch stimulus.^32^ Alike to the MN connections of the BAT-PMRs, the masseteric spindles are also assumed to be monosynaptic in their connection with the trigeminal motor nucleus to expect almost simultaneous latency and action for these two types of BAT-contact-elicited reflexes. It is plausible that the physical distraction of the ipsilateral side by the first BAT-contacts of the BF-bites (Fig. 1, right panel) also causes an ipsilateral jaw-jerk reflex. The observation of the present study of a faster FTt of the BF-bites than of the AF-bites, should reflect of synergy at least, rather than conflict, between the ipsilateral BAT-MN PMR-connections and the spindle-elicited reflexes.

**Fig. 4:**
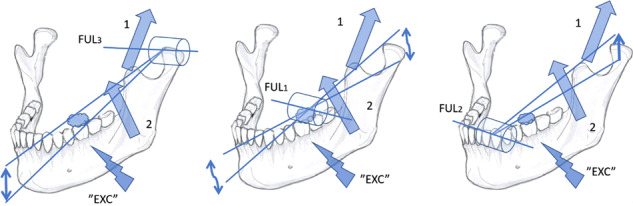
The classes 3 (left), 1 (middle) and 2 (right) lever systems of the vertical phase of closure of the human unilateral chewing cycle. The food bolus is located at the left first molar. The fulcrums corresponding for the class 3, 1 and 2 levers (the FUL3, FUL1 and FUL2, respectively) are encircled. The large arrows designate the active force vectors of the temporal (1) and masseter muscles (2). Instantaneous changes in the resiliency of the food bolus determines the power of the class 1 lever (middle). The class 1 lever system may cause distraction of the FUL3, or hasten the tipping of the anterior part of mandible for ANT contacts.

During the vertical phase of the closure of the human chewing cycle the food items in the BAT-area are first crushed by the class 3 lever (Fig. 4, left panel). The compacting bolus in the BAT-area distracts and shifts the fulcrum inferiorly in the glenoid space of the condyle ipsilateral to the bolus. The compacting, ever denser food bolus also tends to transform into a novel fulcrum for a class 1 lever (Fig. 4, middle panel). Thus, both the fulcrums of class 3 and 1 lever systems are shifting unstably and conflicting with each other. The muscle force is not inhibited. On the contrary, the ongoing unstable stretching of BAT-PMRs ensures rapid excitation of vertical bite force.^8–10^

Chewing food in the BAT-area, the synchronous BAT-MN-inputs and the spindle-mediated ipsilateral jaw-jerk reflex contributes to the rapid “proprioceptive prelude”, the almost immediate activation of the temporal-masseter complex. The anterior part of the mandible is accelerated upwards by the class 1 levered tilt to create the stabilising but inhibitory ANT-contacts. Some dozens of ms later, after the ANT-contact-elicited inhibitory reflexes have waned, the temporalis-masseter complex resumes its activity to cause the “masticatory burst proper”, delivered by the superseding mechanically more advantageous class 2 lever system for which the novel and more rigid ANT-contact is the fulcrum (Fig. 4, right panel).

Likewise, biting on an unexpectedly encountered hard object within a food-bolus in the BAT-area should reinstate the class 1 lever-system victorious, to engage the “proprioceptive prelude” to tilt the tip of the mandible upwards for inhibitory ANT-contacts to protectively stall the chewing process.

Grigoriadis & Trulsson^1^ have demonstrated a stall of masseteric EMG-activity to coincide approximately with the tooth-food contact of the human chewing cycle. They speculated the chewing-induced variations in the duration of the stall of the “bi-phasic” pattern of activation of the masseter muscle might be due to modulation of the CNS-programmed selective activation of muscles. The findings of the present study suggest an alternative explanation for alternating duration of the “stall” between the two “phases” of masseteric activity: the “ANT is not BAT-dichotomy” and the reciprocity of the immediate reflexes they elicit. Chewing food, the food bolus contacts the BAT-area causing the excitatory reflex (phase one), that would be rapidly overrun and stalled by the ANT-contact-elicited inhibitory reflex (the stall), followed by the masseteric force resuming after the ANT-inhibitory reflex (phase two).

The reciprocal reflex-control hypothesis also explains the jaw-unloading reflex—the slow mode of chewing the carrot. When the carrot was placed between upper and lower human incisors, in the absence of excitatory BAT-contacts, the bite force was setting on slowly, inhibited by the ANT-contacts at first. The ANT-PMRs were forced unmovable, since further stretching was prevented by the constant pressure of the carrot against the ANT. The vertical muscle force, the “proper burst”, was finally activated to break down the carrot after a delay of dozens of ms, after the original inhibitory reflex had waned. At the sudden disintegration of the carrot, the instantaneous relief of the incisor loading once again stretched the ANT-PMRs to cause a protective stall of the temporal-masseter complex, after a latency of 6.5–11 ms.^33^

### Anatomical differences of the TMJ between primates and carnivores

Several hypotheses have been discussed to explain the biological significance of the resiliency and the translating abilities of the primate TMJs, that results in the anteroposterior and superoinferior dispositions of the axis of rotation of the condyles during the open-close movements of the mandible.^34,35^ It may be suggested that the resilient translatory abilities of the primate TMJ essentially assist in the proprioception of the mandible-maxilla relation for the mechanically more advantageous class 2 levered chewing force. In comparison, the mandibular condyles of the *Carnivora* are snugly confined within their narrow TMJ-synovial spaces^36,37^ making rigid rotational fulcrums for rapidly deployed, class 3 levered bite forces. The kinematics of human mandible, as observed by the T-Scan, suggests the voluminous synovial fluid compartments of the translating mandibular condyles are a necessity for the adjustment of the maxilla-mandible relation to secure a non-tilting triangle of support to enable the mechanically more advantageous class 2 lever of mandible. The formation of the triangle of support of tooth-food-contacts appears to be the important, but somewhat slow “proprioceptive prelude”, to precede the forthcoming “proper burst” of masticatory muscle force. For primates, it takes more time to create the triangle of support (the STt parameter of this study), whereas the almost non-translating TMJs of most carnivorous mammals are the necessary prerequisite for the immediate class 3 delivered force to puncture the skin of their prey with their prominent canine teeth. Consequently, the bipedal hominin lineage, with their slowly adapting, resilient TMJs, were deemed to evolve their hands and thumbs (and perhaps the brain also) to catch their food and prey.^38^

### The horizontal phase of jaw-closure in mastication

**Fig. 5:**
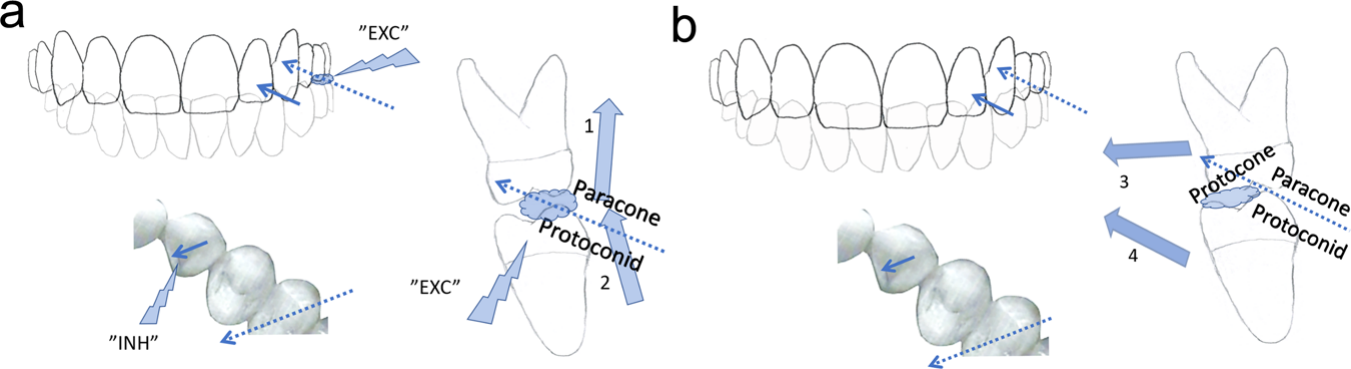
The evolutionally unique human mastication. **a** Selective co-ordination of jaw-adductor muscles in the horizontal phase of closure of the human unilateral chewing cycle. Frontal view (top left), frontal view of a transverse section of the left first molars (right) and an inferior view of the upper left premolars and the canine (left, below). Food-tooth contacts of the BAT-area (“EXC”) activates the temporal (large arrow, 1) and masseter (large arrow, 2) muscles. With the narrowing gape the upper and lower canines are also contacted. In the BAT-area, the lingual inclines of the paracones, and the buccal inclines of the protoconids are guiding the movement of mandible to be approximately parallel (dashed arrow) to the path of the lower canine against the lingual slope of the ipsilateral upper canine (solid arrows). The gliding, inhibitory ANT-contact silences (“INH”) the activity of the temporal-masseter-complex. **b** The medial glide of mandible continues to be driven by the antagonist, horizontally oriented group of muscles, the inferior belly of the lateral pterygoid (large arrow, 3), and the medial pterygoid muscles (large arrow, 4). The food bolus is crushed in the central fossae of the BAT between the buccal aspect of the protocone and the lingual aspect of the protoconid, that are almost perpendicular to the paracone-protoconid-canine-guided medial movement of mandible.

The “reciprocal reflex-control” -hypothesis also explains the selective coordination of jaw-muscles during the transition from the vertical to the horizontal phase of closure of the human chewing cycle. The unilateral food-BAT-contacts are the primary source of excitatory BAT-inputs that boost the temporal-masseter-complex for the vertical crushing movement, with the class 3 or class 1 lever systems (Fig. 5a, top left). Guided by the cuspal inclines of the BAT, the original, more or less vertical path of mandible takes a turn to a more horizontal, medial direction,^13–15^ in parallel to the lingual slopes of the paracones and the buccal slopes of the protoconids of the BAT (Fig. 5 a right), that are also aligned with the lingual slope of the ipsilateral upper canine (Fig. 5a, left, below). The paralleling inclines of the paracone, protoconid and the canine determine the “*angulos funcionales masticatoriós de Planas*”,^39^ the “functional angles of mastication”. In naïve, yet unworn dentitions of adolescents, the paracone-protoconid-canine parallel normally forms an angle of over 30 degrees to the horizontal plane of occlusion, but the functional angle of mastication flattens out with the increasing age, because of physiological wear of teeth.^39^

**Fig. 6:**
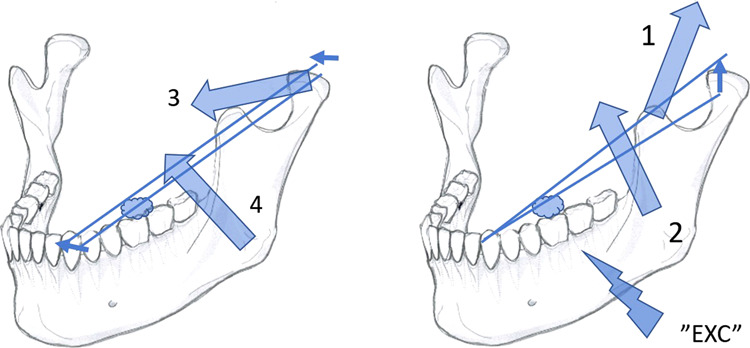
The intermitting bout of vertical muscle force during the horizontal closure phase of the human unilateral chewing cycle. The left panel shows the medial movement of mandible (small, horizontal arrows), driven by the horizontal muscle complex (large arrows, 3 and 4). The food bolus becomes condensed in the first molar area and possibly distracts the ipsilateral synovial space. The right panel illustrates excitatory inputs from the BAT-area (“EXC”). The temporal-masseter-complex (large arrows, 1 and 2) is instantaneously re-activated to crush the bolus vertically (small arrow) with the class 2 lever system. The diminished bolus allows, once again, an increase of the inhibitory ANT-contacts to reinstate the activity of the antagonist, horizontal complex of muscles and the mandible continues to move medially.

During the vertical chewing stroke, the narrowing gape compresses the food bolus in the BAT-area, until the inhibitory ANT-reflex from the contacting, ipsilateral canines inhibits the temporal-masseter complex. The agonist of the vertical phase of closure silenced its antagonist, the horizontally oriented complex of muscles takes over. The activated inferior belly of the lateral pterygoid muscle and the medial pterygoid muscle provide muscle force for the continuation of the medially directed movement of mandible.^16–18^ The food bolus now becomes laterally compacted against the almost perpendicular, opposite slopes of the central fossae of the bilophodont BAT (Fig. 5b). Thus, the medially directed, forceful grinding (Fig. 6, left panel) compacts the food bolus to increase the excitatory BAT-inputs. In addition, the ipsilateral glenoid area becomes distracted to (Fig. 4, middle) to cause spindle-mediated excitatory inputs of the vertical complex of muscles. As result, the temporal-masseter-complex is cyclically activated, over-and-over-again for supplementary coups of class 2 levered vertical force amidst the horizontal grinding process (Fig. 6, right panel).

### The evolutionally unique human mastication

The human mastication enables efficient muscle-driven horizontal grinding with intermitting bouts of vertical force subjected to the entire BAT-area, whereas apes and monkeys seem to protrude their jaw slightly and use their ANT only for the horizontal grinding of food. Their relatively large ANT commonly exhibit edge-to-edge contacts and significant age-related wear, whereas the original inclines of the cusps and fossae of the BAT, except for the C/P3 honing complex, are generally well discernible after decades of arduous chewing of fibrous food.

**Fig. 7:**
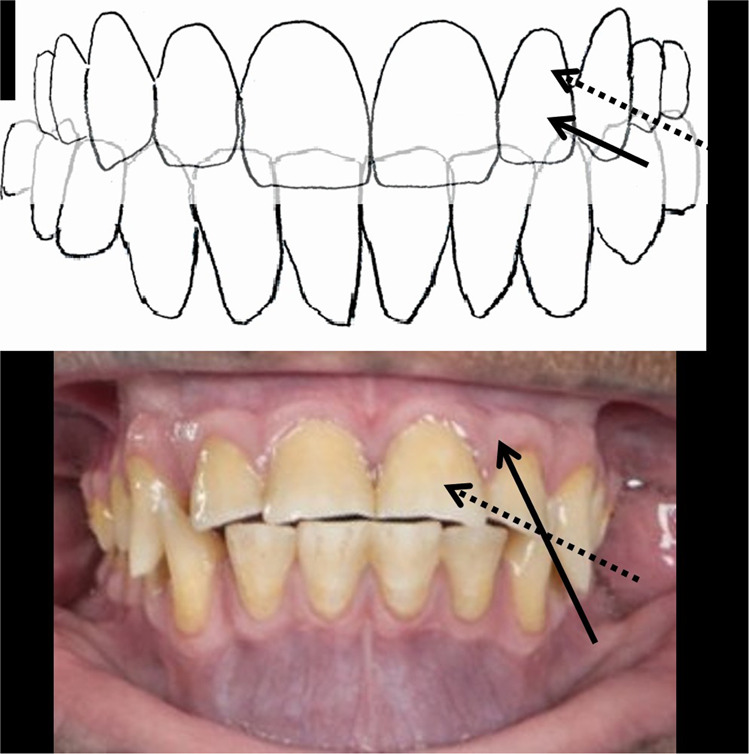
The “reduced canine” -trait that distinguishes humans from other simians. Unlike humans (top), the paralleling cuspal inclines of the paracone/protoconid (dashed arrows) are not equal to the guiding incline (solid arrows) of the large and overlapping upper and lower canines of e.g. *Pan* (below).

The non-human hominids and cercopithecoids share the 2123 dental formula (two incisors, one canine tooth, two premolars and three molars) and the translating TMJs, the combination of which seems to be necessary for the efficient class 2 leverage of vertical bite force to the BAT-area. The horizontal phase of the closure of apes and monkeys is guided by their large and overlapping canines. The distal aspect of the lower canine glides in a steep angle against the mesial slope of the upper canine. The guiding slopes of the contacting upper/lower canines for e.g. *Pan troglodytes* are not parallel to the paracone/protoconid -slopes (Fig. 7). Their BAT-contacts are immediately discluded in the horizontal chewing movements and eliminate their excitatory inputs. The steep canine-guided disclusion of the BAT of the apes and monkeys prevents muscle-driven horizontal food grinding in the BAT-area. The inhibitory inputs of ipsilateral upper and lower canines also silence the temporal-masseter-complex to prevent the BAT-area horizontal grinding of minute seeds and tendinous meats forcefully, as shown for e.g. *Papio anubis.*^40^

Despite the slender and “gracile” features of the bony structures of the human face our chewing capacity is well at par with the much more robustly built other simians^41^ because of the hominin innovation, the reduced canine. The functional demands of the unique hominin type of mastication should exclude the possibility of sexual dimorphism of the human canine despite the dimorphism of canines being prevalent in other simians and our earliest ancestors. The mutation of the MYH16 myosin gene of our ancestors probably coincided with the reduction of the canine.^42^ Thence, the consecutive reduction of the masticatory muscle force might not have been a problem for the hominins in possession of a mechanically more advantageous chewing apparatus. The trade-off for humans with the more advanced leverage of bite force may be the more pronounced physiological wear and flattening of the paralleling inclines of the canines and the guiding paracone/protoconid slopes of the BAT, the functional angles of mastication. The relatively thicker enamel in human molars as compared to *Pan*^43^ probably compensates the age-related wear of human enamel.

Unlike our hunter-gatherer ancestors, contemporary humans are not challenged by hours of masticatory efforts daily. The large horizontal strains of the BAT-area in the human chewing pattern, however, have always challenged the dental profession. The forces subjected to teeth and dental restorations can’t be precisely anticipated by the use of dental articulators. The instantaneous and unforeseen shifts of the location of the resilient fulcrums of classes 3 and 1 lever systems of the chewing force can’t be predicted.

The aetiology of dental bruxism, the excessive wear of teeth, that consecutively causes alterations to the anatomic shape of the mandibular condyles and dental arches is much debated. Dental bruxism may be partly a sleep-related disorder, or psychogenic, nevertheless, there are no reports of this dysfunctional ailment in any non-human simians. An innovative treatment modality employing the T-Scan data to identify the flattened canine-canine guiding angle and to steepen the angle in relation to the guiding parallel of the paracone/protoconid^44^ has been proven successful for many patients suffering from jaw-muscle dysfunctions.^45^

### The tasks of the miniature three ANT of carnivorous mammals

The “canine” of the human dental formula, the third tooth from the midline, is a misnomer. The human canine appears to be wired to stall the temporal and masseter muscles, acting unlike the fangs of the genus *Canis*. The canines of most of the *Carnivora* are the fourth teeth, counting from the midline, and they appear equipped with similar rapid excitatory qualities as the rabbit molar^4^ and the human BAT. The three miniature incisors of the cat and dog dental formulas seem too rudimentary in size to assist significantly in the chewing of food. Perhaps, the inhibitory wiring of the three most anterior teeth of the “generalised mammalian jaw” is a general apomorphy that has evolved to serve as a sensor of tongue-thrusting. The three miniature incisors of the *Carnivora* may be needed to inhibit the temporal and masseter muscles at the initiation of the cascade of the swallowing reflexes, similar to the function of the human incisors and canines combined, the human ANT.^46^

## Conclusions

The present study offers a novel perspective to explain the selective coordination of jaw-muscles in mastication. The almost equally rapid rise of the response of the inhibitory ANT-contact-elicited reflex as compared to the onset of the excitatory sensory inputs from the BAT enables two qualitatively opposite reciprocal reflex systems that determine the selective activation of antagonistic vertical and horizontal jaw-muscles. The findings of this study explain the mechanism by which the instantaneously changing, and unforeseen changes of the force leverage systems of mastication, as presented in Figs. 1 and 4 are coherently responded to. The reciprocity of ANT vs. BAT determining the “fast” and “slow” -modes of mastication, the “central-pattern-generator” -hypothesis of stereotyped CNS-programmed cascades of selectively activated jaw-muscles^47,48^ may be challenged.

The hypothesis presented here and the T-Scan method for its verification provides the basis to explain clinical problems related to prosthodontic treatment planning and solving TMJ-dysfunction issues. Viewed from the “ANT is not BAT” and the “reciprocal reflex-control” -perspectives the different anatomical forms of teeth and dental arches with different dental formulas, as well as the functional anatomy of the TMJs have evolved to conform to the pattern the classes 3, 2 or 1 leverage of masticatory force is used. Understanding the underlying control mechanism of jaw-closures also helps to identify the cladistic nodes of diversifying masticatory functions in the general phylogeny of living and extinct taxa.
